# Supplementary material for: Improving landscape inference by integrating heterogeneous data in the inverse Ising problem
Source: Sci Rep. 2016 Nov 25;6:37812. doi: 10.1038/srep37812 (PMC5122905; doi:10.1038/srep37812)
Supplement: Supplementary Information [file srep37812-s1.pdf]

Supplementary information

Improving landscape inference by integrating heterogeneous data in the inverse Ising problem

Pierre Barrat-Charlaix, Matteo Figliuzzi, and Martin Weigt

Details of the biological data

The following datasets are used in the analysis of biological data:

- **Multiple sequence alignements** We used multiple sequence alignments (MSA) of sequences belonging to the Pfam Beta-lactamase2 family PF13354 for the inference of TEM-1 mutational landscape (resp. PDZ domain family PF00595 for PSD-95 mutational landscape) [30]. Raw data have been processed as described in [18]: we have used HMMer [29] to search against the Uniprot protein sequence database (version of March 2015); from the resulting alignments we have removed all sequences with more than 5 gapped positions; in order to take into account phylogenetic correlations and sampling biases we have associated to each sequence $\boldsymbol{s^m}$ the following statistical weight:

$$w_m = \left(1 + \sum_{m \neq m'} \Theta\Big(d_{m,m'} - \vartheta N \Big) \right)^{-1} , \tag{1}$$

  with $d_{mm'}$ being the Manhattan distance (number of mismatches) between sequences $\boldsymbol{s^m}$ and $\boldsymbol{s^{m'}}$ and Θ being the Heaviside step function whose value is zero for negative argument and one for positive argument. The reweighting threshold is set to $\vartheta = 0.8$ as usually done in DCA inference [32]. The resulting MSA are $N = 197$ (resp. $N = 81$) sites long, and contain $M = 2462$ (resp. $M = 3287$) homologous sequences (average sequence identity $\sim 20\%$ with the wild-type sequences).

- **Computational predictions of thermodynamic stabilities** We used $\Delta\Delta G$-predictions for all single amino acid mutations from the wild-type sequences as estimated by PoPMuSic web-based tool [31] taking as reference structure the PDB: 1M40 for TEM-1 (resp PDB: 1BE9 for PSD-95).

- **Experimental fitness measurements**
  - **TEM-1.** Experimental measurements from [23] provide estimations of amoxicillin Minumum Inibitory Concentration MIC for $n = 742$ distinct single amino-acid mutations falling inside the part of the sequence covered by the corresponding Pfam model. Following [18] we have defined the experimental fitness as the logarithmic average over all MIC measurements of a specific mutant sequence (whenever multiple measurements for the same sequence were available).
  - **PSD-95.** In [22] the effect of each single point mutations has been quantified as the log frequency of observing each amino acid β at each position i in the selected (sel) versus the unselected (unsel) population, relative to amino acid s_i present in the wild type:

$$\phi(s_i \to \beta) = \log\left[\frac{f_i^{sel}(\beta)}{f_i^{sel}(s_i)}\right] - \log\left[\frac{f_i^{unsel}(\beta)}{f_i^{unsel}(s_i)}\right] \tag{2}$$

Potts Hamiltonian

The relevant description in the statistical modeling of protein sequences is a 21-state Potts Model, since each variable $s_i, i = 1, ..., N$, can now assume 21 states (20 amino acids, one alignment gap). The following Potts Hamiltonian provides the energy of any amino-acid sequence $\boldsymbol{s} = (s_1, ..., s_N)$,

$$\mathcal{H}^{\text{Potts}}(\boldsymbol{s}) = -\sum_{i<j}^{N} J_{ij}(s_i, s_j) - \sum_{i=1}^{N} h_i(s_i) \ , \tag{3}$$

where fields h_i and couplings J_{ij} are now respectively 21-dimensional vectors and 21×21-dimensional matrices. We fix the gauge freedom (cf. [1]) using, for all i, j, the lattice-gas conditions $h_i(s_i^{ref}) = 0$ and $J_{ij}(s_i^{ref}, s_j) = J_{ij}(s_i, s_j^{ref}) = 0$. Here $\boldsymbol{s}^{ref} = (s_1^{ref}, ..., s_N^{ref})$ is the reference wildtype sequence, for which mutations shall be predicted.

Fitness-energy mapping

Since model energies $\mathcal{H}(\boldsymbol{\sigma}^{\boldsymbol{a}})$ can be in any non-linear relation with measured fitnesses ϕ^a we replace Eq. (9) with:

$$\begin{aligned} p_{i,\alpha}(\mathbf{J}, \mathbf{h}) &= \pi_{i,\alpha}^{\text{emp}} + \frac{\lambda}{1-\lambda} \frac{1}{M} \sum_{a=1}^{P} \delta_{\sigma_i^a, \alpha} \left[\phi^a - \mu\big(\mathcal{H}(\boldsymbol{\sigma}^a)\big) \right] \\ p_{ij,\alpha\beta}(\mathbf{J}, \mathbf{h}) &= \pi_{ij,\alpha\beta}^{\text{emp}} + \frac{\lambda}{1-\lambda} \frac{1}{M} \sum_{a=1}^{P} \delta_{\sigma_i^a, \alpha} \delta_{\sigma_j^a, \beta} \left[\phi^a - \mu\big(\mathcal{H}(\boldsymbol{\sigma}^a)\big) \right] \end{aligned} \tag{4}$$

where $p_{i,\alpha}(\mathbf{J}, \mathbf{h})$ (resp. $\pi_{i,\alpha}^{\text{emp}}$) are the single marginals in the model (resp. in the empirical sample) specyfing the frequency of observing amino-acid α at position i, $p_{ij,\alpha\beta}(\mathbf{J}, \mathbf{h})$ (resp. $\pi_{ij,\alpha\beta}^{\text{emp}}$) are the pairwise marginals, and $\mu(x)$ is the robust monotonous non-linear mapping from energy to fitness introduced in [18]. The mapping is obtained by first sorting the model energies and experimental fitnesses and then associating the n_{th} smallest energy $\mathcal{H}(n_{th})$ with the n_{th} highest experimental fitness value $\phi(n_{th})$,

$$\mu\big(\mathcal{H}(n_{th})\big) = \phi(n_{th}) \ . \tag{5}$$

Note that the second terms in Eq. (4) vanish when model energies are exactly in the inverse order of experimental fitnesses. We subsequently used the mapping to compute linear correlations between the mapped energies $\mu\big(\mathcal{H}(\boldsymbol{\sigma}^a)\big)$ and the experimental fitnesses ϕ^a, resulting in non-linear rank correlations between experimental fitnesses and model energies.
